# Overexpression of Forkhead Box Protein M1 (FOXM1) in Ovarian Cancer Correlates with Poor Patient Survival and Contributes to Paclitaxel Resistance

**DOI:** 10.1371/journal.pone.0113478

**Published:** 2014-11-20

**Authors:** Fung Zhao, Michelle K. Y. Siu, LiLi Jiang, Kar Fai Tam, Hextan Y. S. Ngan, Xiao Feng Le, Oscar G. W. Wong, Esther S. Y. Wong, Ana R. Gomes, Laura Bella, Pasarat Khongkow, Eric W-F Lam, Annie N. Y. Cheung

**Affiliations:** 1 Department of Pathology, The University of Hong Kong, HKSAR, China; 2 Department of Surgery and Cancer, Imperial College London, London, United Kingdom; 3 Obstetrics and Gynaecology, The University of Hong Kong, HKSAR, China; 4 Department of Pathology, West China Hospital, Sichuang University, Chengdu, China; 5 Department of Experimental Therapeutics, The University of Texas MD Anderson Cancer Center, Texas, United States of America; 6 Department of Pathology, The University of Hong Kong –Shenzhen Hospital, Shenzhen, China; Cincinnati Children's Hospital Medical Center, United States of America

## Abstract

**Aim:**

Deregulation of FOXM1 has been documented in various cancers. The aim of this study was to evaluate the role of FOXM1 in ovarian cancer tumorigenesis and paclitaxel resistance.

**Experimental Design:**

Expression of FOXM1 was examined in 119 clinical samples by immunohistochemistry and correlated with clinicopathological parameters. Effects of FOXM1 knockdown on ovarian cancer cell migration, invasion and mitotic catastrophe were also studied. qPCR and ChIP-qPCR were used to establish KIF2C as a novel FOXM1 target gene implicated in chemoresistance.

**Results:**

High nuclear FOXM1 expression in ovarian cancer patient samples was significantly associated with advanced stages (*P* = 0.035), shorter overall (*P* = 0.019) and disease-free (*P* = 0.014) survival. Multivariate analysis confirmed FOXM1 expression as an independent prognostic factor for ovarian cancer. FOXM1 knockdown significantly inhibited migration and invasion of ovarian cancer cells and enhanced paclitaxel-mediated cell death and mitotic catastrophe in a p53-independent manner. Bioinformatics analysis suggested a number of potential transcription targets of FOXM1. One of the potential targets, KIF2C, exhibited similar expression pattern to FOXM1 in chemosensitive and chemoresistant cells in response to paclitaxel treatment. FOXM1 could be detected at the promoter of KIF2C and FOXM1 silencing significantly down-regulated KIF2C.

**Conclusion:**

Our findings suggest that FOXM1 is associated with poor patient outcome and contributes to paclitaxel resistance by blocking mitotic catastrophe. KIF2C is identified as a novel FOXM1 transcriptional target that may be implicated in the acquisition of chemoresistance. FOXM1 should be further investigated as a potential prognostic marker and therapeutic target for ovarian cancer.

## Introduction

Ovarian cancer is the most lethal gynaecological cancer worldwide as most patients, when diagnosed, are presented with advanced disease [1]. Although improvement in median survival has been observed in recent decades, relapse and mortality rates remain high due in part to the acquisition of chemoresistance [2]. A combination of paclitaxel and carboplatin has widely been used as the first-line chemotherapy for ovarian cancer patients. Paclitaxel acts specifically during the G2-M phase of the cell cycle by inducing abnormal spindles and disruption of microtubule dynamics, thereby blocking cell cycle progression. Despite its initial effectiveness as a cancer therapeutic agent, in most cases, patients eventually become insensitive to paclitaxel-based chemotherapy and relapse. It is therefore vital to identify novel prognostic markers and therapeutic targets, particularly genes related to metastasis and drug resistance.

Forkhead box (FOX) proteins belong to a superfamily of evolutionarily conserved transcription factors responsible for the spatio-temporal fine-tuning of a broad repertoire of transcriptional programmes which are required for the normal homeostasis and development [3]. Among which, forkhead box protein M1 (FOXM1) participates in a wide range of biological processes including cell proliferation, cell cycle progression, cell differentiation, DNA damage repair, tissue homeostasis, angiogenesis and apoptosis [4], [5]. It is therefore not surprising that deregulation of FOXM1 could result in severe pathological conditions, including cancer. Indeed, FOXM1 overexpression has been documented in cancers of the lung, breast, liver, prostate and colon, etc. suggesting that FOXM1 has a key role in tumorigenesis [3], [6]. Recently, it has been shown that deregulated FOXM1 expression can confer resistance to chemotherapeutic drugs, such as cisplatin and epirubicin, by protecting cells against DNA-damage induced cell death, and disrupting the mitotic checkpoint [7]–[9].

In this study, we investigated the expression pattern of FOXM1 in ovarian cancer. The effects of FOXM1 expression on cancer cell migration, invasion and paclitaxel resistance were also studied in an attempt to evaluate FOXM1 as a potential molecular prognostic marker and therapeutic target for ovarian cancer. Further analyses were performed to identify KIF2C as a novel FOXM1 transcriptional target that might be implicated in the acquisition of chemoresistance.

## Materials and Methods

### Clinical samples and cell lines

Archival paraffin embedded tissue blocks from year 1987 to 2004 were retrieved from the Department of Pathology, Queen Mary Hospital, the University of Hong Kong. The samples included 2 benign cystadenomas, 2 borderline tumours, 94 primary carcinomas and 21 metastatic foci of cancer (at ligament, gut, lymph node and uterine serosa). All patients with carcinoma underwent surgery followed by the standard first-line chemotherapy including platinum/paclitaxel. The follow-up period ranged from 5 to 209 months (median 63 months). The use of these samples was approved by the Institutional Ethical Review Board. Each patient sample was assessed by pathologists and ensured to contain more than 70% tumour cells.

Ovarian cancer cell lines SKOV-3 and OVCAR-3 were purchased from American Type Culture Collection (Manassas, VA, USA). SKOV3-TR cells were a generous gift from Dr Lawrence XF Le (Division of Cancer Medicine, University of Texas M.D. Anderson Cancer Center, Houston, Texas, USA) [10]. PEO1 and PEO1-TaxR are recently developed cell lines and have been authenticated at Cancer Research UK facility. The use of PEO1 and PEO1-TaxR instead of SKOV-3 and SKOV3-TR for some experiments offers extra means to ensure that resistant mechanisms identified in SKOV-3 and SKOV3-TR are common to all paclitaxel resistant ovarian cancer cells and not unique to SKOV-3 and SKOV3-TR. Importantly, both PEO1 and PEO1-TaxR are kept to lower passages to avoid drifts in resistance and the acquisition of secondary mutations [10] [11]. OVCAR-3 was cultured in 1∶1 Medium 199 (Invitrogen, CA, USA): MCDB105 (Sigma, MO, USA) supplemented with 10% foetal bovine serum (FBS) and 100 units/ml penicillin-streptomycin (Invitrogen). SKOV3, SKOV3-TR, PEO1 and PEO1-TaxR were cultured in RPMI1640 (Sigma) supplemented with 10% foetal bovine serum (FBS) and 100 units/ml penicillin-streptomycin (Invitrogen). PEO1-TaxR was supplemented with 50 nM paclitaxel. All cell lines were maintained at 37°C in humidified incubator with 5% CO_2_. Cell culture medium was changed every 3 to 5 days depending on cell density. For routine passage, when cells reached 85% to 90% confluency, they were split at a ratio of 1∶4.

### Immunohistochemistry

Immunohistochemistry was performed as described previously [12], [13]. Briefly, formalin-fixed paraffin sections were incubated with anti-FOXM1 antibody (NBP1-30961, 1∶40, Novus Biologicals, CO, USA) at room temperature overnight and stained using EnVision+ Dual Link System (K4061; Dako, CA, USA). Antigen retrieval was performed using EDTA buffer, pH8.0, in a pressure cooker for 30 min. All sections were assessed by two independent investigators. The immunoreactivity of FOXM1 antibody, the intensity of stained cells and their percentages were measured in terms of intensity and percentage scores respectively. The percentage score ranged from 0 to 4: 0 = <5% of positively stained cells, 1 = 5–25% of positively stained cells, 2 = 26–60% of positively stained cells, 3 = 61–85% of positively stained cells, and 4 = 86–100% of positively stained cells. Immunohistochemical (IHC) score (from 0 to 16) was calculated by multiplying the intensity score (0–4) and the percentage score (0–4), with a maximum score of 16 [12], [13]. FOXM1 nuclear and cytoplasmic immunoreactivities were scored separately.

### Western blot

Cells were harvested with lysis buffer [0.125 m Tris, pH 6.8 at 22°C containing 1% NP-40 (v/v), 2 mM ethylenediamine tetraacetic acid (EDTA), 2 mM N-ethylmaleimide, 2 mM phenylmethanesulphonyl fluoride (PMSF), 1 mM sodium orthovanadate and 0.1 µm sodium okadate] and centrifuged at 4°C for 10 min. Protein concentration was determined by detergent-compatible (DC) protein assay (Bio-Rad). Twenty micrograms of protein were separated by sodium dodecyl sulphate-polyacrylamide gel electrophoresis (SDS-PAGE), transferred to polyvinylidene difluoride membrane and hybridized with the following anti-bodies: anti-FOXM1 (sc-502, Santa Cruz Biotechnology, CA, USA), anti-Caspase 9 (9502, Cell Signaling Technology, MA, USA), anti-KIF2C (WH0011004M1, Sigma), anti-Caspase 7 (9492, Cell Signaling) and anti-β-tubulin (sc-9104, Santa Cruz Biotechnology).

### Quantitative real-time PCR

Quantitative real-time PCR (qPCR) was performed using Power SYBR Green PCR Master Mix (Applied Biosystems) and analysed by ABI7900 Sequence Detection System (Applied Biosystems). L19 (RPL19), a non-regulated ribosomal housekeeping gene was used as an internal control for normalization using the delta-delta Ct method. The following primers were used:

KIF2C Forward 5′ to 3′: CATGATTGCCACGATCTCAC

KIF2C Reverse 5′ to 3′: CGTTAGAGCAGGCTTCCATC

L19 Forward 5′ to 3′: GCGGAAGGGTACAGCCAAT

L19 Reverse 5′ to 3′: GCAGCCGGCGCAAA

### Transient knockdown of FOXM1

ON-TARGET Plus Human FOXM1 siRNA and Non-targeting Control siRNAs (Thermo Scientific, CO, USA) were employed for transient silencing of FOXM1 using Oligofectamine transfection reagent (Invitrogen) as per the manufacturer's instructions.

### *In Vitro* migration and invasion assays

*In Vitro* migration and invasion assays were performed as described previously [12], [13]. Briefly, 1.25×10^5^ cells were plated on the upper compartment of a Transwell chamber (Corning Life Sciences, MA, USA). For migration assays, cells were allowed to migrate through a gelatin-coated membrane. For invasion assays, cells were allowed to invade through a matrigel-coated membrane. After 24 h, cells on the upper side of the membrane were removed and the migrated or invaded cells were fixed, stained and counted.

### TdT-mediated dUTP nick end labeling (TUNEL) assay and evaluation of mitotic catastrophe index

Following FOXM1 knockdown for 48 h and paclitaxel treatment (50 nM) for 24 h, TUNEL assay was performed using In Situ Death Detection Kit (Roche Biochemical, IN, USA) following the manufacturer's protocol [14]. Apoptotic and mitotic catastrophe figures were assessed under fluorescence microscopy. Mitotic catastrophe figures were observed by morphological changes in nuclei (DAPI staining) [10]. More than 1000 viable cells in each experiment were examined and the mitotic catastrophe index was evaluated as percentages of the cells counted. Every assay was run in triplicate.

### Cell cycle analysis

Cell cycle analysis was performed by propidium iodide staining as described previously [15]. Briefly, both adherent and suspension cells were harvested and stained with propidium iodide (1 mg/mL) in the presence of DNase-free RNase for flow cytometric analysis. Cell cycle profile was analyzed by using the Cell Diva software (Becton Dickinson UK Ltd.).

### Chromatin Immunoprecipitation

40 µl of Dynabeads Protein A (10002D, Invitrogen) was washed with 200 µl of TSE I buffer for three times and diluted with 40 µl of TSE I buffer. Anti-FOXM1 (sc502, Santa Cruz Biotechnology) (4 µg) and rabbit IgG control (X0903, DAKO) (4 µg) were first separately diluted in Buffer D, mixed with diluted Dynabeads and then rotated O/N at 4°C. PEO1 and PEO1-TaxR cells at 90% confluency in 100 mm culture dish were crosslinked with 1% formaldehyde for 10 min, rinsed with ice-cold PBS and incubated with 2.5 M glycine for 5 min. Cells were then harvested with 2 ml of scrapping buffer. After a sequential wash with PBS, Buffer I and Buffer II, cell pellet was resuspended in 300 µl of Lysis buffer and subjected to sonication under optimized condition (20 min with 30 s on and 30 s off). Supernatant was then diluted in 300 µl of Buffer D from which 100 µl was taken as INPUT control. 200 µl of cell lysate was mixed with prepared Dynabeads and rotated O/N at 4°C. After a sequential wash with TSE I, TSE II, Buffer III and TE buffer, 100 µl of elution buffer was added to the Dynabeads and the mixture was rotated at RT for 1 h. Eluted sample was collected in eppendorf and the Dynabeads was re-eluted with another 100 µl of elution buffer. 200 µl of sample was de-crosslinked by incubating at 65°C O/N. PCR Purification Kit (Qiagen) was then used to purify DNA. Quantitative real-time PCR was performed with the following primers: KIF2C (Forward 5′ to 3′: GCCAAGTCTCCAACTTGCTC; Reverse 5′ to 3′: TTCCCAACCATCTTCCTACG).

ChIP-qPCR data was normalized to IgG control and plotted as percent input. Composition of buffers was listed in Table 1.

**Table 1:** Composition of buffers used in ChIP.

Buffer	Composition
Buffer I	0.25% Triton X-100, 10 mM EDTA, 0.5 nM EGTA, 10 mM HEPES pH6.5
Buffer II	200 mM NaCl, 10 mM EDTA, 0.5 mM EGTA, 10 mM HEPES pH6.5
Buffer III	0.25 M LiCl, 1% NP-40, 1% deoxycholate, 1 mM EDTA, 10 mM Tris-HCL pH8.1
Lysis buffer	1% SDS, 10 mM EDTA, 50 mM Tris-HCL pH8.1
Buffer D	1% Triton X-100, 2 mM EDTA, 20 mM Tris-HCL pH8.1, 150 mM NaCl
TSE I	0.1% SDS, 1% Triton X-100, 2 mM EDTA, 20 mM Tris-HCL pH8.1, 150 mM NaCl
TSE II	0.1% SDS, 1% Triton X-100, 2 mM EDTA, 20 mM Tris-HCL pH8.1, 500 mM NaCl
TE buffer	10 mM Tris-HCL pH8.0, 1 mM EDTA
Scrapping buffer	100 mM Tris-HCL pH9.4, 0.1% SDS
Elution buffer	0.1 M NaHCO_3_, 1% SDS

### Statistical analysis

Statistical analysis was performed by the Statistical Package for Social Science version 19.0 for Windows (IBM). Comparison between two groups of non-parametric data was performed by Mann-Whitney *U*-test. Probability of survival was analyzed using the Kaplan-Meier approach. Multivariate analysis of prognostic factors was performed using Cox's regression model. *P*-values of <0.05 were considered to be statistically significant.

## Results

### FOXM1 overexpression correlates with poor survival

**Figure 1:**
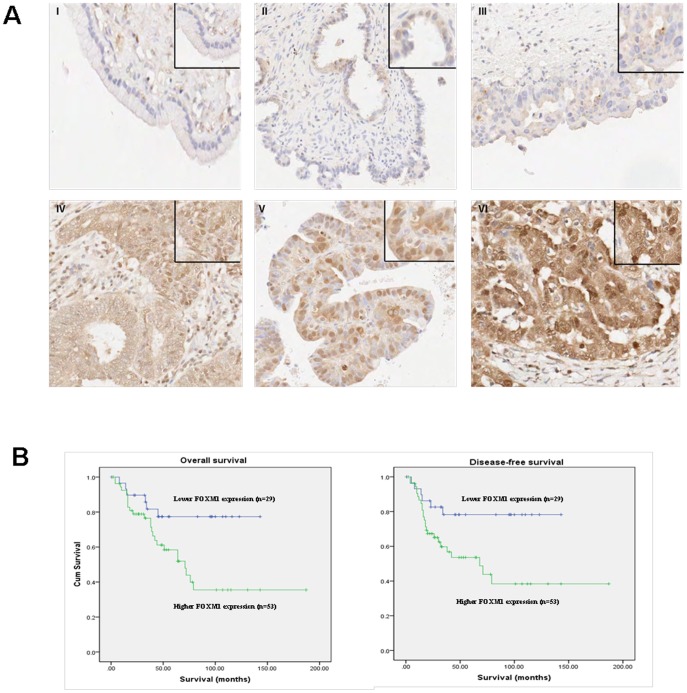
Elevated nuclear FOXM1 expression was associated with advanced stages of ovarian cancer. **A**, Representative images of immunoreactivity of nuclear FOXM1 in (I) benign cystadenoma, (II) borderline tumour, (III) stage I invasive cancer, (IV) stage II invasive cancer, (V) stage III invasive cancer and (VI) stage IV invasive cancer. Magnifications X400. Insets: Regions with higher magnifications of nuclear FOXM1 staining. **B**, Cumulative overall and disease-free survival plots using the Kaplan-Meier approach.

The expression of nuclear FOXM1 in ovarian benign and borderline tumours as well as invasive cancers was evaluated by immunohistochemistry. Ovarian cancers displayed stronger nuclear FOXM1 staining than benign and borderline tumours. However, there was no significant difference in FOXM1 expression between primary carcinomas and their metastatic foci (*P* = 0.6). Higher nuclear FOXM1 expression was significantly associated with advanced stages of ovarian cancer (*P* = 0.035) (Fig. 1A). Although not reaching statistical significance, FOXM1 overexpression displayed a trend related to serous histological type (*P* = 0.142), high grade cancers (poor differentiation) (*P* = 0.235) and chemoresistance (*P* = 0.282) (Table 2). In order to study the association of FOXM1 expression with patients' outcome, patients were categorized into two groups using the cutoff point of IHC score>0. As shown in the Kaplan-Meier overall survival plot (Fig. 1B), patients with negative FOXM1 staining had a significantly longer overall (*P* = 0.019) and disease-free survival (*P* = 0.014) than those with positive FOXM1 expression. Multivariate progression analysis showed high expression of FOXM1, advanced cancer stages and poor histological differentiation (high grade) were found to be independent prognostic factors for short overall survival (95% CI, 0.906–5.754, HR 2.283, *P* = 0.08; 95% CI, 0.953–5.963, HR 2.384, *P* = 0.06; 95% CI, 2.137–40.638, HR 9.318, *P* = 0.003, respectively) and disease-free survival (95% CI, 1.025–6.631, HR 2.607, *P* = 0.04; 95% CI, 1.039–6.555, HR 2.61, *P* = 0.04; 95% CI, 1.821–35.091, HR 7.993, *P* = 0.006, respectively). Interestingly, cytoplasmic staining of FOXM1 was also detected in addition to nuclear expression, but no significant correlation with clinicopathological parameters was observed (data not shown).

**Table 2:** Summary of nuclear FOXM1 immunohistochemical staining results in various types of ovarian tumors.

		Nuclear expression of FOXM1					
		Absent (IHC = 0)	Weak (IHC = 1–3)	Moderate (IHC = 4–6)	Strong (IHC = 7–12)	Total	p-value
DiagnosticCategories							
	Benign	1 (50%)	1 (50%)	0 (0%)	0 (0%)	2	
	Borderline	0 (0%)	2 (100%)	0 (0%)	0 (0%)	2	
	Invasive cancer	31 (33%)	28 (29.8%)	25 (26.6%)	10 (10.6%)	94	
	Metastatic foci	3 (14.3%)	8 (38.1%)	6 (28.6%)	4 (19%)	21	
Histologic Types							
	Mucinous	3 (33.3%)	4 (44.4%)	1 (11.1%)	1 (11.1%)	9	
	Serous	11 (31.4%)	7 (20%)	11 (31.4%)	6 (17.1%)	35	0.142*
	Endometrioid	14 (45.2%)	10 (32.3%)	7 (22.6%)	0 (0%)	31	
	Clear cell	4 (21.1%)	7 (36.8%)	5 (26.3%)	3 (15.8%)	19	
Stages							
	Stage I	15 (41.7%)	11 (30.6%)	9 (25%)	1 (2.78%)	36	
	Stage II-IV	12 (24%)	13 (26%)	16 (32%)	9 (18%)	50	0.035†
Grades							
	Grade I	10 (41.7%)	8 (33.3%)	5 (20.8%)	1 (4.17%)	24	
	Grades II–III	22 (31.9%)	19 (27.5%)	19 (27.5%)	9 (13%)	69	0.235‡
Chemosensitivity							
	Sensitive	28 (41.2%)	14 (20.6%)	18 (26.5%)	8 (11.8%)	68	
	Resistant	3 (17.6%)	8 (47.1%)	3 (17.6%)	3 (17.6%)	17	0.282Υ

*p value reflects the comparison of serous ovarian cancer vs. all the other histological types combined.

† p value reflects the comparison of stage I vs. stage II–IV.

‡ p value reflects the comparison of low grade (grade I) vs. high grade (grade II and III).

Υ p value reflects the comparison of chemosensitive vs chemoresistant cases.

### Transient knockdown of FOXM1 inhibits SKOV-3 cell migration and invasion

**Figure 2:**
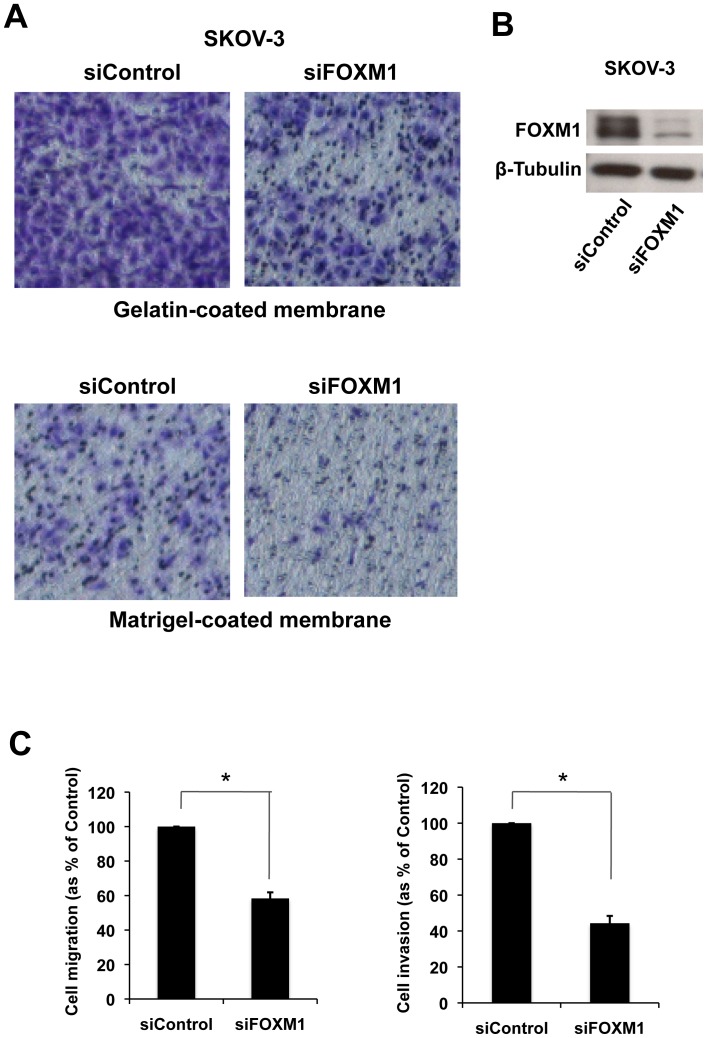
Silencing of FOXM1 reduced migration and invasion of SKOV-3. **A**. Representative images showing cells migrated (gelatin-coated membrane) or invaded (matrigel-coated membrane) after 24 h. **B**. Representative Western blot analysis demonstrating the effectiveness of FOXM1 transient knockdown in SKOV-3. **C**. Graphic representation of migration (left panel) and invasion (right panel) results as fold change of migrated and invaded cells relative to the control, respectively, in five fields of triplicate wells from three independent experiments; * P<0.05, significant; Mann-Whitney *U*-test.

We next studied the role of FOXM1 in ovarian cancer progression. *In Vitro* Transwell assays were employed to study the effects of transient silencing of FOXM1 on ovarian cancer cell motility and invasion. Significantly decreased migration and invasion (*P*<0.05) was observed in SKOV-3 cells transfected with ON-TARGET Plus Human FOXM1 siRNA (siFOXM1) as compared to cells transfected with control siRNA (siControl), indicating that knockdown of FOXM1 was capable of inhibiting migration and invasion of SKOV3 cells (Fig. 2).

### Paclitaxel treatment downregulates the expression of FOXM1 in SKOV-3 but not in the paclitaxel-resistant SKOV3-TR

**Figure 3:**
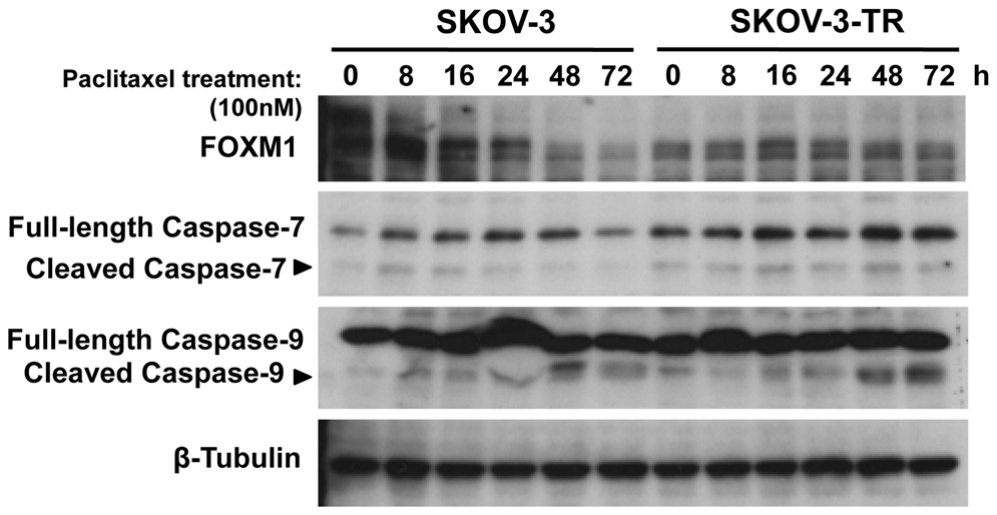
Paclitaxel treatment down-regulates FOXM1 expression in SKOV-3 but not in SKOV3-TR cells. The paclitaxel sensitive SKOV-3 and resistant SKOV3-TR ovarian cancer cells were treated with 100 nM paclitaxel and harvested at times indicated for Western blot analysis. Paclitaxel treatment down-regulated FOXM1 expression at time points 48 h and 72 h in SKOV-3 but not in SKOV3-TR as shown by immunoblotting. There were also no marked changes in the cleaved Caspase-9 and Caspase-7 expression.

In view of the IHC staining showing that elevated FOXM1 expression is associated with poor prognosis and thus chemoresistance, a pair of established ovarian cancer cell line sensitive and resistant to paclitaxel, namely SKOV-3 and SKOV3-TR respectively, were used to study the effect of paclitaxel treatment on the expression of FOXM1. Cells were treated with paclitaxel (100 nM) and harvested at various time points 0, 8, 16, 24, 48 and 72 h. Intriguingly, immunoblotting showed FOXM1 expression to be decreased at 48 h and 72 h in SKOV-3. However, FOXM1 expression remained relatively constant at high levels in SKOV3-TR upon paclitaxel treatment (Fig. 3), suggesting a role of FOXM1 in mediating paclitaxel resistance in ovarian cancer cells. Notably, there also appeared to be only marginal increases in the expression of cleaved Caspase-9 and Caspase-7 in both SKOV-3 and SKOV3-TR upon paclitaxel treatment, suggesting that apoptosis may not be the predominant mechanism of inducing cell death in these ovarian carcinoma cells.

### Transient silencing of FOXM1 significantly enhances paclitaxel-mediated mitotic catastrophe

**Figure 4:**
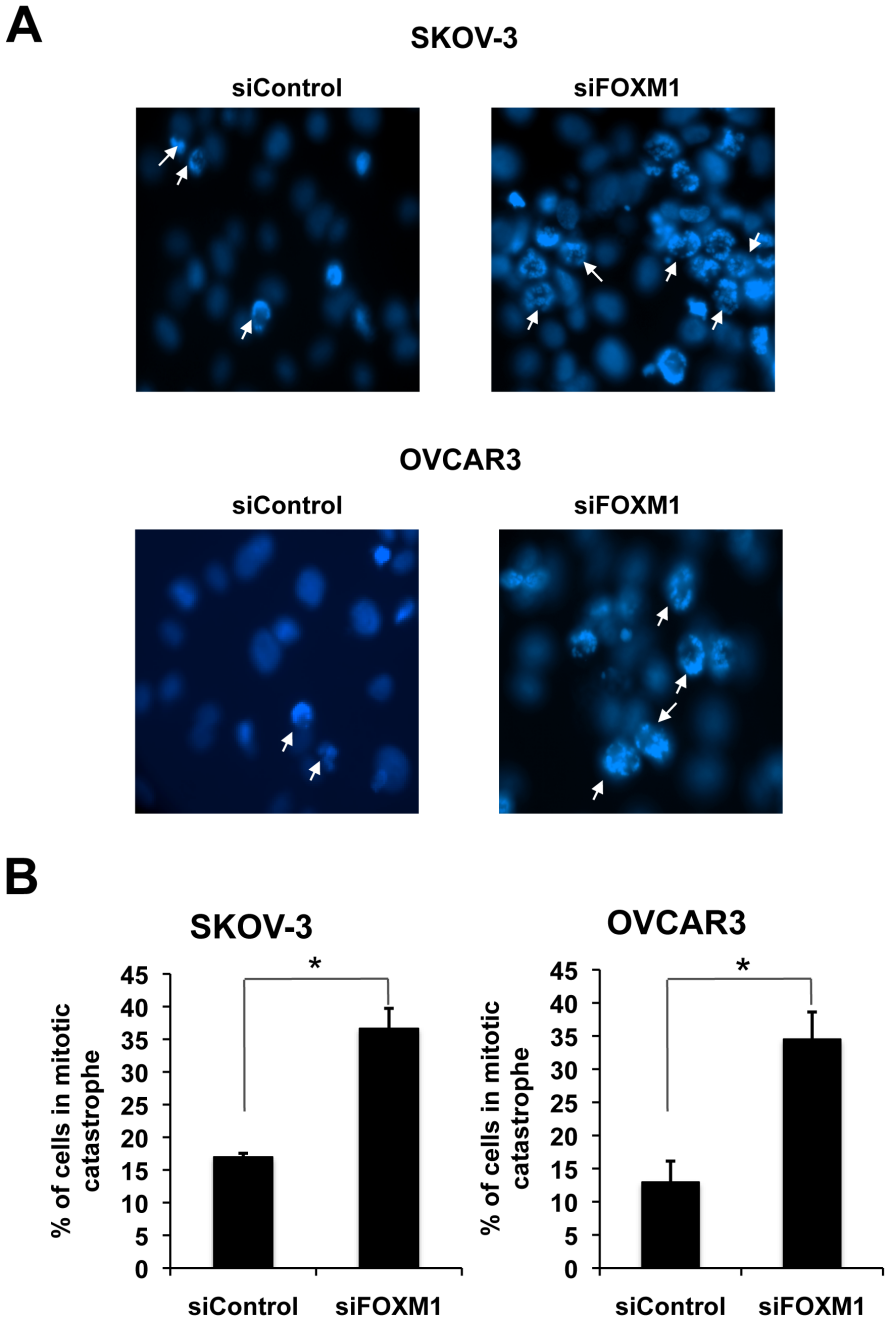
Transient FOXM1 knockdown significantly enhanced paclitaxel-induced mitotic catastrophe in SKOV-3 and OVCAR3 cells. SKOV-3 and OVCAR3 cells transfected with either siRNA pools against FOXM1 or control siRNA pools were treated with paclitaxel (100 nM) and stained with DAPI. **A.** Representative staining results were shown showing that transient FOXM1 knockdown significantly enhanced paclitaxel-induced mitotic catastrophe (arrow) in SKOV-3 and OVCAR3 cells respectively (Upper panel). **B**. Graphs represent the results of three independent experiments, showing the percentage of cells undergoing mitotic catastrophe, * P<0.05, significant; Mann-Whitney *U*-test.

We previously showed that paclitaxel kills ovarian cancer cells predominantly by inducing mitotic catastrophe rather than apoptosis in the cells [10]. To elucidate the potential role of FOXM1 in ovarian cancer chemoresistance, SKOV-3 and OVCAR3 ovarian cancer cell lines were treated with paclitaxel for 24 h following FOXM1 depletion by siRNA, stained with DAPI and examined by fluorescent microscopy. The result showed there was an increased number of multi-nucleated cells (arrow) in ovarian cancer cells with FOXM1 knockdown (*P* = 0.03 and 0.01, respectively) (Fig. 4), suggesting FOXM1 depletion significantly enhanced paclitaxel-mediated mitotic catastrophe in both SKOV-3 and OVCAR3. It is notable that apoptosis was barely detectable in these paclitaxel-treated cells. This was probably due to the fact that both SKOV-3 and OVCAR3 harbour dysfunctional p53, and functional p53 is required for paclitaxel-induced apoptosis in ovarian cancer cells.

### FOXM1 knockdown induces modest increases in the accumulation of G2/M and dead cells upon paclitaxel treatment in the resistant SKOV3-TR cell line

**Figure 5:**
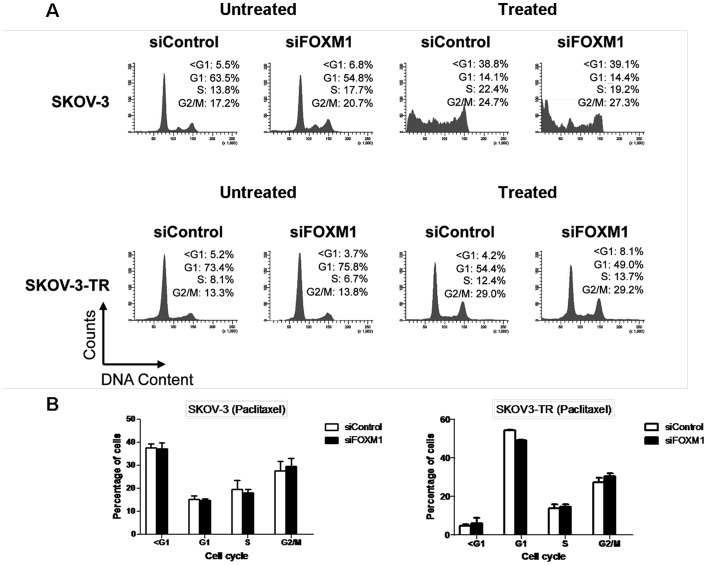
Flow cytometric analysis of SKOV-3 and SKOV-3-TR cells with and without FOXM1 depletion in the presence or absence of paclitacel treatment. **A**. Flow cytometric analysis was performed following propidium iodide staining on SKOV-3 and SKOV-3-TR cells treated with paclitaxel (100 nM) or remained untreated after transfection with siRNA pools against FOXM1 or control siRNA pools. Representative data are shown indicating that FOXM1 silencing is capable of increasing the number of dead cells and cells blocked at G2/M cell cycle phase in SKOV-3-TR as compared to cells treated with control siRNA. **B**. Bar charts of different phases of cell cycle in SKOV-3 and SKOV3-TR treated with paclitaxel after transfection with control or siRNA against FOXM1. Results represent data from two independent experiments.

Cell cycle analysis was then performed to elucidate the role of FOXM1 in paclitaxel-mediated cell death. To this end, SKOV-3 and SKOV3-TR cells were transiently transfected with control and FOXM1 siRNA for 48 h and cultured in the presence or absence of paclitaxel treatment (100 nM) for 48 h. The resultant cells were harvested, stained with propidium iodide and subjected to flow cytometric analysis. The results showed that paclitaxel induced significant levels of cell death (sub-G1 population) in SKOV-3 cells transfected with either FOXM1 or control siRNA pool (Fig. 5A, upper panel). Increased number of cells accumulated with sub-G1 DNA contents in SKOV3-TR with FOXM1 knockdown (8.1%) (Fig. 5A, upper panel) compared to SKOV3-TR transfected with control siRNA (4.2%) (Fig. 5A, upper panel), suggesting FOXM1 contributes to paclitaxel resistance in ovarian cancer cells and that FOXM1 silencing can enhance paclitaxel-mediated cell death. Upon paclitaxel treatment, a modest increase in cells blocked at G2/M phase was also observed in SKOV3-TR treated with siRNA against FOXM1 as compared to control, suggesting FOXM1 silencing might enhance paclitaxel-mediated cell death via mitotic catastrophe (Fig. 5A, lower panel; Fig. 5B). Depletion of FOXM1 in the sensitive SKOV-3 cells has no additive effect to paclitaxel treatment. This is likely due to the fact that paclitaxel functions through downregulating FOXM1 expression as revealed by the Western blot analysis (see Fig. 3).

### KIF2C is identified as a novel FOXM1 transcriptional target that may be implicated in the acquisition of chemoresistance

**Figure 6:**
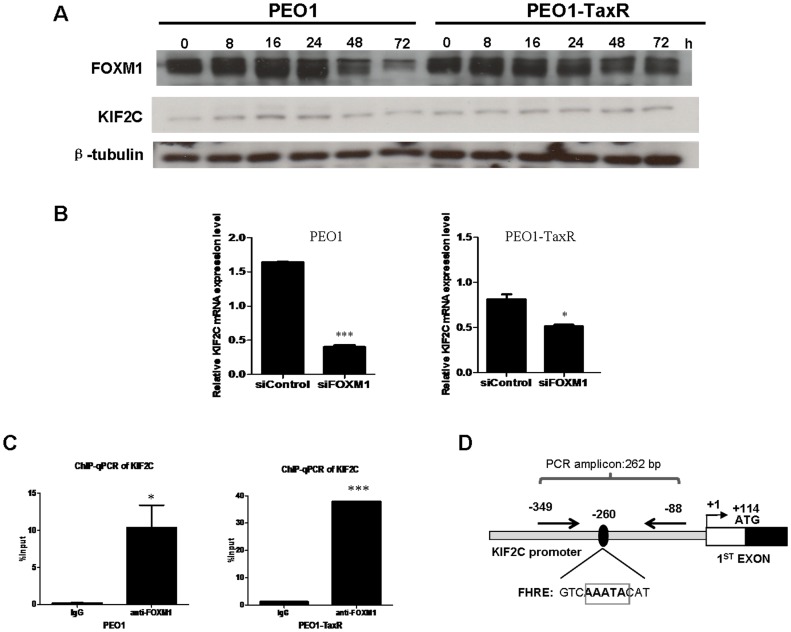
Identification of KIF2C as a novel FOXM1 transcriptional target that might be implicated in the acquisition of paclitaxel resistance. **A**, Paclitaxel treatment (50 nM) down-regulated FOXM1 and KIF2C expressions at 48 h and 72 h in PEO1 but not in PEO1-TaxR. **B**, FOXM1 knockdown significantly reduced the transcript level of KIF2C in PEO1 and PEO1-TaxR. Data represent triplicates from three experiments. *P = 0.04, ***P = 0.0003. siControl: Non-specific control. siFOXM1: FOXM1 knockdown. **C**, ChIP-qPCR showed FOXM1 significantly pull down KIF2C promoter region in PEO1 and PEO1-TaxR as compared to the negative IgG control. Data represent triplicates from three experiments. *P = 0.04, ***P = 0.0001. **D**, Schematic diagram depicting locations of forkhead response element (FHRE) and the binding site of primers used in ChIP-qPCR upstream of the transcription start site of KIF2C.

Bioinformatics analysis revealed KIF2C, a member of the KIF superfamily of proteins, as a potential target gene of FOXM1 with consensus forkhead binding sites located upstream of the transcription start site (Fig. 6D). Using a pair of paclitaxel-sensitive (PEO1) and -resistant (PEO1-TaxR) ovarian cancer cell line, paclitaxel treatment down-regulated the expression of KIF2C at 48 h and 72 h in PEO1. However, KIF2C expression remained relatively constant in PEO1-TaxR upon paclitaxel treatment (Fig. 6A), suggesting a role of KIF2C in mediating paclitaxel resistance in ovarian cancer cells. Intriguingly, the expressions of FOXM1 changed in a similar pattern (Fig. 6A). Quantitative real-time PCR (qPCR) was then used to study the effect of transient silencing of FOXM1 on the transcript level of KIF2C. FOXM1 knockdown resulted in significantly down-regulated mRNA expressions of KIF2C in both cell lines (P<0.05) (Fig. 6B). Chromatin immunoprecipitation-qPCR (ChIP-qPCR) further showed FOXM1 was capable of binding to the promoter region of KIF2C (P<0.05) (Fig. 6C), implying KIF2C might serve as a novel FOXM1 transcriptional target.

## Discussion

In this study, we show that high nuclear FOXM1 expression is significantly correlated with stage, shorter overall survival and disease-free survival of ovarian cancer patients. Multivariate analysis indicates that FOXM1 expression could serve as an independent prognostic factor. Furthermore, transient FOXM1 depletion is capable of inhibiting ovarian cancer cell migration. These findings suggest that FOXM1 can have a crucial role in ovarian carcinogenesis and progression and may predict patients' outcome.

Although FOXM1's association with high grade ovarian carcinomas has been reported [16], whether FOXM1 participates in the acquisition of paclitaxel resistance remains undefined. Indeed, deregulated FOXM1 expression has been shown to confer resistance to chemotherapeutic drugs such as cisplatin and epirubicin [8], [9]. In view of the immunohistochemical finding suggesting association between FOXM1 and chemoresistance, a pair of established paclitaxel-sensitive and -resistant cell lines, SKOV-3 and SKOV3-TR [10], were employed to study the effect of paclitaxel on FOXM1. Interestingly, paclitaxel treatment resulted in down-regulation of FOXM1 in SKOV-3 but not in the resistant cell line SKOV3-TR, implying a role of FOXM1 in mediating paclitaxel resistance in ovarian cancer cells. Immunofluorescence study further showed transient FOXM1 knockdown could enhance paclitaxel-mediated cell death in two ovarian cancer cell lines, SKOV-3 (deleted p53)[17] and OVCAR3 (mutant p53) [18]. It is not surprising that apoptotic cells were barely detectable as both cell lines harbour dysfunctional p53. Recently, it was suggested that the induction of p53-independent apoptosis takes place through the activation of Caspase-9 [19]. However, immunoblotting revealed Caspase-9 was not activated upon paclitaxel treatment in SKOV-3 and SKOV-3-TR cells, indicating that both paclitaxel and FOXM1 silencing effect cell death primarily through enhancing mitotic catastrophe rather than apoptosis in ovarian cancer cells, which commonly have dysfunctional p53 pathway. This is consistent with previous findings on paclitaxel in breast cancer [20].

Mitotic catastrophe can be considered as a type of cell death occurring during mitosis or resulting from mitotic failure [21]. Two mechanisms have been suggested as crucial for mitotic catastrophe, namely the G2/M and mitotic spindle checkpoints [22]. For the G2/M checkpoint, the inactivation of genes such as *p53*, *p21^Cip1^* and *14-3-3Sigma* have been reported to induce DNA damage-induced mitotic catastrophe [23], [24]. Flow cytometric analysis performed in our study suggested FOXM1 knockdown in the chemoresistant ovarian cancer cell line SKOV3-TR could induce cell death. Paclitaxel treatment and immunofluorescent analysis further suggested FOXM1 silencing could enhance paclitaxel-mediated mitotic catastrophe in a p53-independent and Caspase-9-independent manner. Delineation of the underlying mechanism by which FOXM1 mediates paclitaxel resistance will shed light on novel approaches of treatment.

Kinesin superfamily proteins (KIFs) play pivotal roles in intracellular transport of organelles and maintenance of spindle assembly during mitosis and meiosis [25]. Being the founding and best-characterized member of the kinesin-13 family, KIF2C/MCAK is crucial for ensuring the faithful segregation of chromosomes in mitosis and for safeguarding chromosomal stability [26]. Not surprisingly, up-regulations of KIF2C have been documented in multiple human cancers and KIF2C has been suggested to play an important role in carcinogenesis [27], [28]. In the current study, immunoblotting analysis showed KIF2C expression in PEO1 altered in a similar pattern as FOXM1 expression by displaying a down-regulation at 48 h and 72 h upon paclitaxel treatment. In contrast, KIF2C expression remained relatively constant in PEO1-TaxR, implicating KIF2C might be involved in the development of paclitaxel resistance in ovarian cancer. This finding is consistent with a recent report demonstrating loss of KIF2C could increase the sensitivity of Chinese hamster ovary (CHO) cells to paclitaxel [29]. Furthermore, KIF2C was identified as a novel FOXM1 transcriptional target that might play a pivotal role in mediating paclitaxel resistance in ovarian cancer cells.

In conclusion, overexpression of FOXM1 was found to be correlated with poor patients' survival and to paclitaxel-mediated mitotic catastrophe in ovarian cancer cells. KIF2C was identified as a novel FOXM1 target gene implicated in the mediation of paclitaxel resistance. Our findings help to define FOXM1 as a potential prognostic marker as well as a therapeutic target in ovarian cancer.
